# IFITM3 at the viral fusion-pore checkpoint: membrane energetics and entry-route-dependent outcomes

**DOI:** 10.3389/fimmu.2026.1928879

**Published:** 2026-08-24

**Authors:** Luyu Mao, Siqi Wang, Yang Bai, Shuchang Cheng, Kaili Gao, Yiming Chang, Lianmin Li, Yongli Guo, Mingchun Gao

**Affiliations:** 1 College of Veterinary Medicine, Northeast Agricultural University, Harbin, Heilongjiang, China; 2 Department of Immunology, School of Basic Medical Sciences, Harbin Medical University, Harbin, Heilongjiang, China

**Keywords:** entry route, fusion pore, hemifusion, host restriction, IFITM proteins, IFITM3, membrane fusion, viral entry

## Abstract

Interferon-induced transmembrane proteins (IFITMs) are small interferon-stimulated membrane proteins that can limit viral entry, but their effects depend on the virus, entry route, and experimental context. This Mini Review focuses on IFITM3 at the viral fusion-pore checkpoint, defined here as the transition at which hemifusion either proceeds to fusion-pore opening and viral-content release or stalls before productive entry. Work on influenza virus supports a model in which IFITM3 remodels late-endosomal membranes, prolongs or kinetically stabilizes hemifusion-like intermediates, and raises the activation-energy barrier to fusion-pore formation. Type I interferon signaling, host biological variation, IFITM3 polymorphisms, and post-translational regulation shape IFITM3 abundance and localization, whereas endolysosomal cholesterol homeostasis modifies the membrane environment encountered by incoming virions; whether altered sterol handling also changes IFITM3 turnover remains unresolved. The fusion-pore checkpoint is presented as a working model supported most directly by mechanistic studies of influenza A virus. Coronavirus studies and a Nipah virus entry model illustrate how IFITM phenotypes can shift among restriction, neutrality, and context-dependent support. We therefore interpret IFITM activity as an entry-route-dependent membrane phenotype and outline practical criteria for assigning these outcomes in viral entry studies.

## 1 Introduction

Viral entry is one of the earliest points at which an infected cell can prevent productive infection. For enveloped viruses, entry is not completed by receptor engagement or endocytosis alone. Viral and cellular membranes must pass through apposition, hemifusion, fusion-pore opening, and viral-content release. Small changes in lipid order, membrane fluidity, curvature, stiffness, or vesicular trafficking can therefore decide whether an incoming virion establishes infection.

IFITM1, IFITM2, and IFITM3 were first defined as interferon-stimulated restriction factors with activity against influenza A virus, West Nile virus, and dengue virus (1). Early mechanistic studies placed IFITM3 at the cytosolic-entry step of influenza virus infection rather than at receptor binding alone (2). The biological importance of this family was further supported by *in vivo* work showing that IFITM3 contributes to host control of influenza A virus infection (3). Subsequent studies extended IFITM activity to several enveloped virus families, including filoviruses, coronaviruses, and flaviviruses (4, 5).

IFITM activity therefore cannot be reduced to a binary sensitive/resistant label. Influenza virus provides the clearest fusion-restriction model, whereas coronaviruses can show restriction, little effect, or context-dependent support depending on cell type, protease usage, IFITM abundance, and entry compartment (6–8). The key question is whether an incoming virion attempts fusion in a membrane compartment physically remodeled by IFITM3. Here, the term fusion-pore checkpoint denotes the transition at which a hemifusion intermediate either progresses to pore opening and viral-content release or stalls, with the viral cargo potentially routed toward degradation.

Broader reviews have addressed viral-envelope determinants, immune regulation, cancer, and viral evasion (9–11). We focus instead on the IFITM3-dependent energetic barrier to fusion-pore formation and on how entry route should guide phenotype assignment. Direct entry-stage evidence is distinguished from conclusions based only on later infection endpoints, particularly when receptor trafficking, protease availability, or cell type can redirect viral entry.

## 2 Localization and structural determinants of IFITM activity

IFITM activity is closely tied to subcellular localization. IFITM1 is found predominantly at the plasma membrane and in early endocytic compartments, whereas IFITM2 and IFITM3 are enriched in endosomal and lysosomal compartments (4, 12). This spatial bias helps explain member-specific differences. A virus fusing at or near the cell surface may encounter IFITM1 more readily, whereas a virus that traffics into late endosomes is more likely to encounter IFITM3. Recent analyses of endogenous proteins further indicate that IFITM1 and IFITM3 can co-reside and cooperate in acidic endolysosomes, with IFITM3 contributing to IFITM1 localization in these compartments (13).

Localization is regulated rather than fixed. IFITM2 and IFITM3 contain a tyrosine-based YxxΦ trafficking motif that supports endolysosomal sorting, whereas IFITM1 lacks this canonical motif. Phosphorylation and ubiquitination of the IFITM3 N-terminal region regulate endocytosis, stability, and antiviral activity (14, 15). Recent work identified MARCH8-mediated K63-linked polyubiquitination at K24 as an additional pathway controlling IFITM3 lysosomal turnover and trafficking (16). Mutating trafficking motifs or forcing IFITM redistribution can convert the apparent phenotype of an IFITM protein, especially in assays using viral glycoproteins that fuse at different cellular sites (6, 17).

The conserved CD225 domain and amphipathic helix provide distinct determinants of IFITM3 activity. The CD225 domain supports IFITM homo-/heteromeric association and antiviral activity against influenza A virus and dengue virus (18). The amphipathic helix influences membrane order, cholesterol interaction, curvature, and fluidity (19, 20, 38). Membrane order, stiffness, and fluidity are related but not interchangeable: order describes lipid packing, stiffness reflects resistance to deformation, and fluidity describes the dynamic mobility of membrane constituents, including their lateral diffusion. Direct measurements of lipid or protein lateral diffusion in native IFITM3-positive endosomal membranes remain limited; most available evidence derives from probes sensitive to membrane order or fluidity and from membrane-mechanical measurements (33, 38). Interferon-inducible phospholipid remodeling has been linked to IFITM3-dependent endosomal antiviral activity (21). At the host-machinery interface, the CD225 domain contains an R-SNARE-like motif through which IFITM3 binds syntaxin 7 (STX7), disrupts assembly of the SNARE complex that drives homotypic late-endosome fusion, and accelerates delivery of endosomal cargo to lysosomes (22). Mutations that abrogate STX7 binding also impair anti-influenza activity, linking endosomal membrane-trafficking control to restriction; however, the extent to which this pathway contributes directly to virus-specific fusion-pore arrest remains unresolved (22).

Post-translational modifications tune this membrane-associated activity. S-palmitoylation increases IFITM3 membrane association and supports restriction (23, 24). ZDHHC20 promotes IFITM3 palmitoylation (25), while sterol interactions and palmitoylation influence antiviral specificity (26). ABHD16A negatively regulates IFITM palmitoylation (27). IFITM3 has also been reported to bind vesicle-associated membrane protein-associated protein A (VAPA) and disrupt its association with oxysterol-binding protein (OSBP), thereby perturbing endosomal cholesterol export and intracellular cholesterol homeostasis (28). This pathway links host lipid-transfer machinery to the membrane environment encountered by entering virions, although it should not be treated as the sole explanation for IFITM3-mediated restriction.

Human IFITM3 genetic variation adds a clinically relevant layer of host heterogeneity. The rs12252-C allele has been associated with severe influenza in some populations, although findings have varied across cohorts and ancestries (29). The regulatory variant rs34481144 has been associated with 2009 pandemic H1N1 infection, whereas rs12252 and related haplotypes have also been examined in COVID-19 cohorts (30, 31). These epidemiological associations do not establish a fusion-stage mechanism without functional evidence linking the variants to IFITM3 abundance, localization, and entry-specific activity.

Dose and expression context also matter. Endogenous IFITM3 abundance is not a fixed background variable; it can differ among tissues, cell types, individuals, and basal interferon states. Physiological interferon induction and plasmid-driven overexpression are not equivalent. Overexpression may drive IFITMs into membranes where they would not normally accumulate, alter receptor distribution, perturb endosomal traffic, or change global membrane order. These caveats are especially relevant in coronavirus models, where overexpressed IFITMs can reduce surface ACE2 availability while endogenous IFITM2/3 may support viral replication in selected epithelial systems (8, 32). Interpretation should therefore begin with endogenous protein abundance and localization rather than with transcript induction or endpoint antiviral activity alone.

## 3 IFITM3 as an energetic barrier to fusion-pore formation

The strongest mechanistic evidence for IFITM restriction comes from influenza virus entry, although early studies did not localize the dominant block identically. Li et al. (33) reported inhibition at or before hemifusion, whereas Desai et al. (34) placed the principal block after lipid mixing but before fusion-pore opening. Differences in assay resolution, viral system, and IFITM expression likely contribute to this discrepancy. Together, these studies position IFITM3 near the transition from hemifusion to productive pore formation rather than at receptor binding or downstream replication.

Live-cell imaging of IFITM3-positive compartments strengthened this compartmental model. Sensitive influenza particles became trapped in IFITM3-containing endosomes, whereas resistant Lassa virus particles could escape such compartments more efficiently (35). IFITM3 can also engage incoming virus particles and promote their trafficking toward lysosomes, providing a possible downstream route for stalled viral cargo (36).

Recent biophysical imaging sharpened the model. IFITM3 localizes to late-endosomal compartments and induces local lipid sorting at virus–endosome contact sites. This remodeling prolongs or kinetically stabilizes hemifusion-like intermediates and is interpreted as raising the activation-energy barrier to fusion-pore formation (37). Viral-content release consequently fails in a proportion of particles, and stalled cargo may subsequently be routed toward lysosomal degradation. Direct binding of IFITM3 to a viral glycoprotein is not required by this model; instead, IFITM3 modifies the target membrane so that pore opening becomes energetically unfavorable.

Complementary membrane-biophysical studies support this interpretation. Cell-based Laurdan measurements associated IFITM expression with increased lipid order and reduced membrane fluidity (33), whereas reconstituted IFITM3 and amphipathic-helix studies demonstrated increased lipid order and stiffness and curvature changes that disfavor productive fusion (38). In HIV-1 producer cells, Nef impaired IFITM3 oligomerization and counteracted the IFITM3-dependent reduction in membrane fluidity, thereby partially restoring membrane permissiveness (50). Together with the VAPA-OSBP and SNARE pathways, this finding illustrates how lipid-transfer machinery, vesicle-fusion machinery, and a viral antagonist can converge on the localization or physical state of IFITM3-positive membranes without implying a single universal mechanism across viruses. Cholesterol binding by the IFITM3 amphipathic helix and sterol-sensitive antiviral activity connect restriction to lipid organization (20, 26). Related entry-restriction pathways, such as CH25H-dependent production of 25-hydroxycholesterol and ZMPSTE24-mediated antiviral defense, further illustrate that host cells can interfere with entry by changing membrane permissiveness (39, 40).

**Figure 1:**
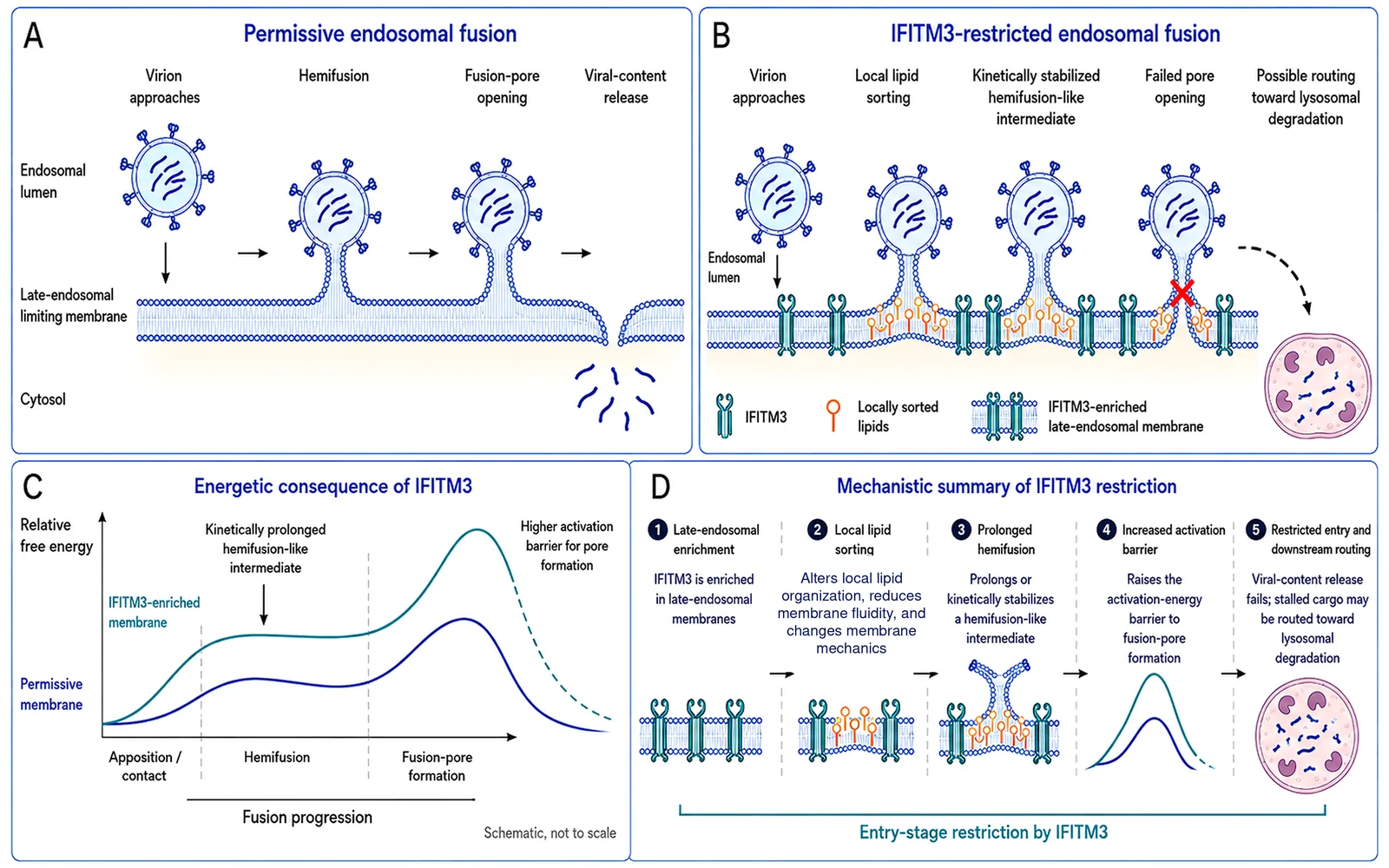
Working model of the IFITM3-dependent energetic barrier to viral fusion-pore opening. **(A)** During permissive endosomal fusion, the viral and late-endosomal membranes progress from apposition through hemifusion and fusion-pore opening, allowing viral-content release into the cytosol. **(B)** In an IFITM3-enriched late-endosomal membrane, local lipid sorting and membrane remodeling prolong a hemifusion-like intermediate and disfavor fusion-pore opening. Stalled viral cargo may subsequently be routed toward lysosomal degradation. The orange symbols represent locally sorted lipids schematically and do not specify cholesterol or another individual lipid species. **(C)** Conceptual free-energy landscape showing a kinetically prolonged hemifusion-like intermediate and a higher activation barrier for fusion-pore formation in an IFITM3-enriched membrane. The dashed teal segment represents a hypothetical post-barrier trajectory. Panel **(C)** is a qualitative conceptual schematic, not a profile generated by molecular-dynamics simulations, potential-of-mean-force sampling, or other quantitative free-energy calculations; the axes and curve magnitudes are not to scale. **(D)** Mechanistic summary linking late-endosomal IFITM3 enrichment, local lipid sorting, prolonged hemifusion, increased resistance to fusion-pore formation, and restricted viral entry with possible downstream lysosomal routing. This working model is supported most directly by mechanistic studies of influenza A virus (34, 36, 37). IFITM3 is depicted schematically, and its icon is not intended to specify a definitive membrane topology. IFITM3, interferon-induced transmembrane protein 3.

The energetic-barrier model explains both broad restriction and its context dependence: a virus that fuses in an IFITM3-rich late endosome may encounter a strong barrier, whereas a virus entering through an IFITM-poor compartment or at the plasma membrane may avoid it. The fusion-pore checkpoint should therefore be treated as a working model: direct mechanistic evidence is strongest for influenza A virus, and extension to other viruses requires entry-stage assays rather than inference from reduced viral load alone. **Figure 1** contrasts permissive and IFITM3-restricted endosomal fusion, presents a conceptual energy landscape, and summarizes the proposed sequence from late-endosomal enrichment to restricted entry and possible lysosomal routing.

## 4 Entry-route-dependent outcomes: restriction, neutrality, and context-dependent support

IFITM phenotypes can be grouped into restriction, neutrality, and context-dependent support. Restriction is best supported when endogenous or near-physiological IFITM expression reduces productive entry or virion infectivity, as seen in influenza virus and several other enveloped-virus systems (1, 33, 37). Neutrality occurs when the viral entry route or cellular context does not place the virus in contact with a restrictive IFITM-modified compartment. Context-dependent support is more difficult to interpret and requires strict controls for expression level, localization, and entry route. These categories are assay-level assignments rather than fixed properties of a virus. The same viral glycoprotein may be sensitive in an endosomal route, less affected at the plasma membrane, or appear supported when receptor trafficking or IFITM abundance has changed. The terms restriction and context-dependent support should therefore be tied to the entry system being tested, not used as permanent labels for a virus–IFITM pair.

Coronavirus entry provides a clear example of this context dependence. SARS-CoV-2 can enter through endosomal cathepsin-dependent pathways or through plasma-membrane fusion when TMPRSS2 is available. Human and mouse IFITM proteins can restrict SARS-CoV-2 infection in some systems, but altered trafficking motifs and plasma-membrane fusion can change the phenotype (6, 7). Other studies reported IFITM-dependent enhancement of SARS-CoV-2 infection or a supportive role for endogenous IFITM2/3 in SARS-coronavirus replication, whereas overexpression inhibited infection by relocalizing ACE2 and reducing surface availability (8, 41). Such supportive effects may occur at or beyond entry and should not be interpreted as direct facilitation of fusion unless entry-stage assays demonstrate this. These observations are compatible if endogenous and overexpressed IFITMs are treated as distinct experimental states rather than interchangeable models.

Earlier human coronavirus work also showed that individual IFITM residues can switch restriction and enhancement during coronavirus entry (17). A Nipah virus entry model provides another example in which IFITM3 promoted envelope protein-mediated entry in a defined MDCK-cell system and interacted with the fusion subunit of the F protein (42). Such observations should not be generalized as broad proviral activity. They show that an IFITM-modified membrane can be restrictive, irrelevant, or permissive depending on glycoprotein requirements, receptor usage, and fusion site.

Viral-envelope properties—including thickness, curvature, glycoprotein density, fusion pH, and plasma-membrane versus endosomal protease requirements—can determine whether an IFITM-modified membrane is restrictive or largely irrelevant (10). For this reason, broad claims that a virus is IFITM-sensitive should specify the glycoprotein, cell type, entry route, protease context, and IFITM member tested.

## 5 Physiological modulators and experimental standards for interpreting entry phenotypes

**Figure 2:**
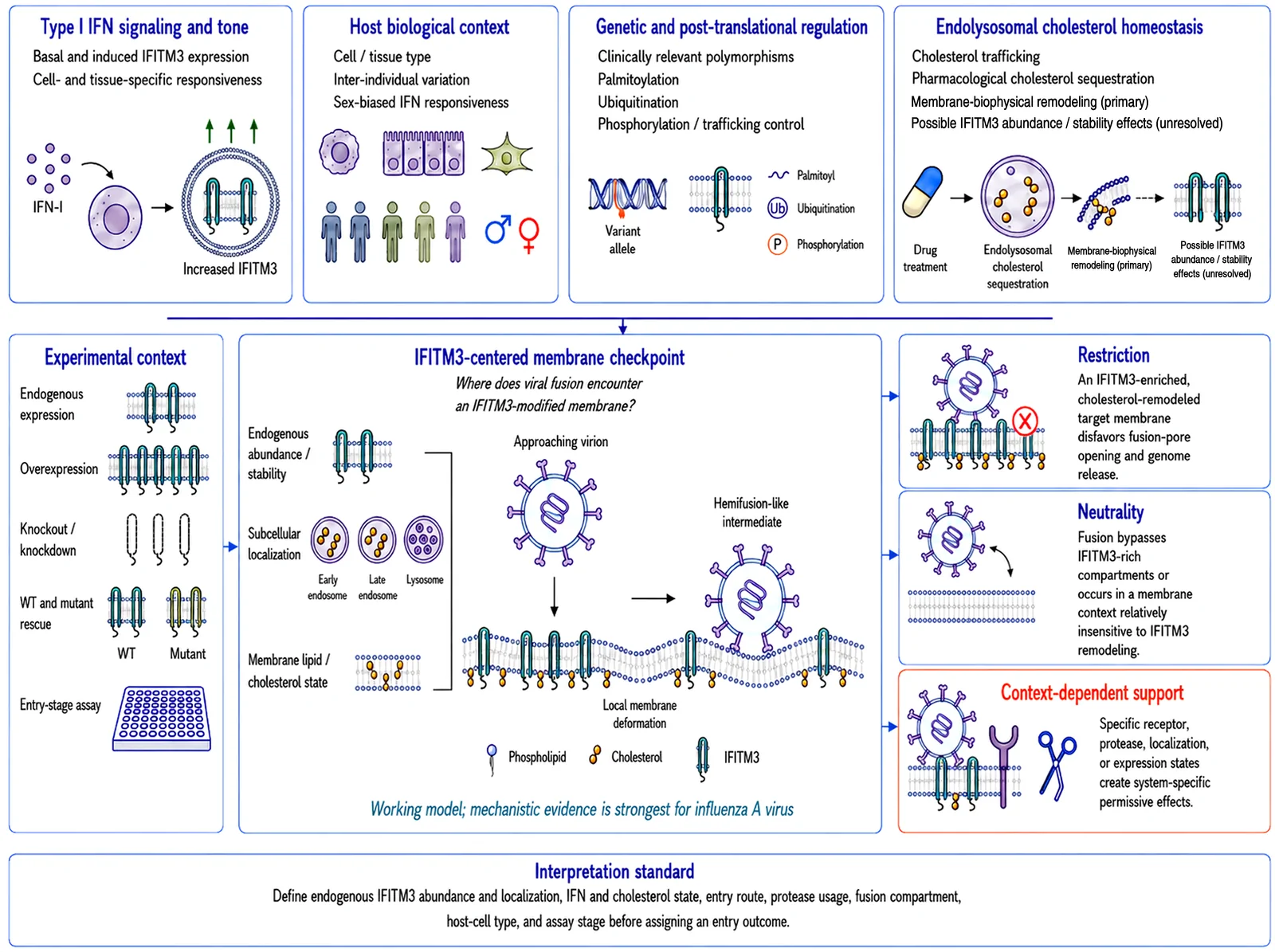
Context framework for IFITM3-dependent viral entry outcomes. Type I IFN signaling and tone, host biological context, and genetic and post-translational regulation shape endogenous IFITM3 abundance, stability, and localization, whereas endolysosomal cholesterol homeostasis is depicted primarily as a modifier of the membrane environment encountered by incoming virions. In the cholesterol module, the solid left-to-right sequence represents drug treatment, endolysosomal cholesterol sequestration, and membrane-biophysical remodeling, including changes in lipid order, fluidity, stiffness, and curvature. The dashed arrow denotes only a proposed, incompletely validated effect on IFITM3 abundance or stability and is not presented as the primary mechanism. Experimental conditions further influence the observed phenotype. The IFITM3-centered membrane checkpoint is a working model supported most directly by mechanistic studies of influenza A virus. Depending on entry route, protease usage, fusion compartment, receptor context, and the IFITM3-modified membrane environment, viral entry may result in restriction, neutrality, or context-dependent support. Solid arrows indicate supported relationships, whereas dashed arrows indicate proposed or incompletely validated links. IFITM3, interferon-induced transmembrane protein 3; IFN, interferon; WT, wild type.

These virus-dependent outcomes are further shaped by physiological factors that determine how much IFITM3 reaches the relevant membrane and in what molecular state. Type I IFN signaling regulates basal and induced IFITM3 expression, but the amount of protein reaching a relevant membrane varies with cell and tissue type, host genotype, inflammatory state, and biological sex. Sex-biased type I IFN responses have been documented across immune settings (43). In plasmacytoid dendritic cells, heterogeneous TLR7 escape from X-chromosome inactivation contributes to this difference (44), while DDX3-dependent regulation provides an additional mechanism (45). These mechanisms may contribute to IFITM3 heterogeneity *in vivo*, but a stronger IFN response does not necessarily imply greater functional IFITM3 abundance at the viral fusion site without direct protein and localization measurements. **Figure 2** integrates these physiological variables with the experimental factors used to assign entry outcomes.

Endolysosomal cholesterol homeostasis can modify both the membrane environment encountered by incoming virions and the cellular response to antiviral drugs. Itraconazole inhibits influenza A virus *in vitro* and *in vivo*, with antiviral activity associated with altered cholesterol homeostasis and interferon priming (46). Itraconazole- or fluoxetine-induced cholesterol imbalance also impairs Ebola virus infection, while fluoxetine perturbs endolysosomal acidification and promotes cholesterol accumulation during SARS-CoV-2 infection (47, 48). These studies support endolysosomes as host-directed antiviral targets (49), although altered acidification, trafficking, and membrane mechanics may act independently of IFITM3. Cholesterol binds the IFITM3 amphipathic helix, and IFITM3 itself perturbs intracellular cholesterol homeostasis (20, 28). Whether altered sterol handling changes IFITM3 abundance or stability remains unresolved. Accordingly, **Figure 2** depicts drug treatment followed by endolysosomal cholesterol sequestration and membrane-biophysical remodeling, including changes in lipid order, fluidity, stiffness, and curvature, with solid arrows. A dashed arrow is reserved for the possible downstream effect on IFITM3 abundance or stability, which is not presented as the primary or an established mechanism.

Physiological variation is difficult to interpret without matched experimental controls. Endogenous IFITM mRNA and protein should be measured under basal, interferon-stimulated, and infected conditions because transcript induction does not establish protein abundance at the fusion membrane. Overexpression should be paired with knockdown or knockout and rescue using wild-type and localization- or modification-defective mutants. Expression control, localization mapping, entry-stage readouts, and genetic rescue in the same system help separate membrane remodeling from receptor redistribution, altered interferon signaling, or later replication effects (32).

Entry-stage resolution is equally important. Attachment, internalization, hemifusion, fusion-pore opening, viral-content release, and downstream replication are distinct events. Single-particle fusion assays, time-of-addition studies, and entry-specific reporters provide stronger evidence than endpoint viral RNA or titer measurements. IFITM localization should be visualized with relevant compartment markers and entry-factor measurements. Receptor and protease context is especially important for coronaviruses, because ACE2 abundance, TMPRSS2 expression, and cathepsin usage can redirect entry and alter IFITM sensitivity.

A claim of direct entry restriction should satisfy four criteria: the protein is expressed at a physiological level; it localizes to the relevant fusion site; loss-of-function and rescue reproduce the phenotype; and the affected step is resolved, distinguishing attachment, internalization, hemifusion, pore formation, and viral-content release from later replication events.

Apparent context-dependent support requires comparable scrutiny. It should be tested against receptor redistribution, membrane composition, protease usage, and IFITM abundance. A supportive phenotype may simply reflect rerouting into a compartment where IFITM-dependent membrane organization no longer poses a barrier.

Quantitative endogenous tagging and matched loss-and-rescue designs are needed to determine IFITM3 abundance at fusion sites and how cholesterol-related changes shift membrane permissiveness. Beyond influenza virus, assays should separate hemifusion, pore opening, viral-content release, receptor trafficking, protease routing, and post-entry effects instead of treating support as a single proviral category. Combining endogenous perturbation with single-particle entry assays and direct membrane readouts should clarify when polymorphisms, tissue-specific expression, or sex-biased IFN states matter at host scale. Functional studies of IFITM3 variants should move beyond association cohorts to endogenous editing in primary airway epithelium, organoids, or other tissue-relevant systems, with matched measurements of protein abundance, localization, and entry-stage function. The relevant question is which conditions make an IFITM3-modified membrane decisive for entry.

## 6 Discussion

IFITM3 provides the best-resolved example of an intrinsic membrane barrier acting near the transition from hemifusion to pore formation. In influenza A virus, late-endosomal IFITM3 alters local lipid organization, prolongs or kinetically stabilizes hemifusion-like intermediates, and raises the activation-energy barrier to pore opening. This working model explains restriction without requiring a shared viral target, but its extension to other viruses requires stage-resolved evidence.

IFITM phenotypes remain conditional. IFN tone, host background, genetic and post-translational regulation, cholesterol homeostasis, and experimental expression determine IFITM3 abundance and membrane localization, while entry route and protease usage determine whether a virus encounters that state. These variables can invert apparent outcomes, especially in coronavirus systems. Mechanistic claims should therefore be based on endogenous abundance, localization, entry-stage measurements, and genetic rescue rather than endpoint infection alone.

At host scale, reduced pore formation could lower the probability that an incoming virion establishes productive infection, but effects on susceptibility, tissue tropism, or disease outcome require direct testing and will also depend on inoculum, viral fitness, and the local tissue environment. Resolving these relationships will require endogenous, stage-resolved measurements in physiologically relevant cell and tissue systems.
